# Draft genome sequences of an *Enterobacter hormaechei* and *Providencia rettgeri* isolated from the urine of a male experiencing a catheter-associated urinary tract infection

**DOI:** 10.1128/mra.00486-24

**Published:** 2024-07-31

**Authors:** Helen Appleberry, Michaela Brady, Samantha Webster, Alan J. Wolfe, Catherine Putonti, Alex Kula

**Affiliations:** 1 Department of Biology, Loyola University Chicago, Chicago, Illinois, USA; 2 Bioinformatics Program, Loyola University Chicago, Chicago, Illinois, USA; 3 Department of Microbiology and Immunology, Loyola University Chicago, Maywood, Illinois, USA; University of Maryland School of Medicine, Baltimore, Maryland, USA

**Keywords:** *Enterobacter hormaechei*, *Providencia rettgeri*, catheter-associated urinary tract infection, CAUTI, urinary tract infection

## Abstract

Catheter-associated urinary tract infections (CAUTIs) can be caused by a variety of microbes. Here, we describe the draft genome assemblies of two species*—Enterobacter hormaechei* and *Providencia rettgeri*—purified from the catheterized urine sample of a male diagnosed with a CAUTI.

## ANNOUNCEMENT

While upward of 70% of catheter-associated urinary tract infections (CAUTIs) are estimated to be preventable (1), they are one of the most common healthcare-associated infections in the United States (2). *Escherichia coli*, *Staphylococcus aureus*, and *Klebsiella* species are the three most frequently reported pathogens, although several other bacterial and yeast species have been identified, including *Enterobacter* species (3). In September 2020, a urine sample was tested by the Loyola University Chicago Clinical Laboratory from a male diagnosed with a CAUTI; this testing identified *Enterobacter* and *Proteus mirabilis* present in the sample. The *Enterobacter* isolate from this urine sample was processed using the expanded quantitative urine culture method (4) and identified by matrix-assisted laser desorption ionization-time of flight (MALDI-TOF) as *Enterobacter cloacae*, as previously described (5). The isolate was stored at −80°C in the Loyola Urinary Education and Research Collaborative (LUEREC) collection. The freezer stock was streaked onto a Columbia naladixic acid agar plate and incubated for 24 hours at 35°C in 5% CO_2_; upon inspection, two distinct colony morphologies were observed. Subsequent rounds of purification were performed using Nutrient Broth plates and media, incubated using the conditions above. Whole-genome sequencing of the purified cultures identified two species: *Enterobacter hormaechei* and *Providencia rettgeri*. In a prior study of *Enterobacter*-associated UTIs, *E. hormaechei* was found to be the most frequent species (6), and misidentification of this species by MALDI-TOF is common (7). Although far less commonly associated with UTIs, cases of *P. rettgeri*-associated UTIs and CAUTIs have been presented in the literature (8, 9).

The original catheterized urine sample was collected from a male donor as part of a prior Institutional Review Board-approved study (LU212677 and LU217801). For each of the purified morphologies, DNA was extracted from the liquid culture using the Qiagen DNeasy Blood and Tissue Kit’s protocol for Gram-positive organisms. DNA was sent to SeqCoast Genomics (Portsmouth, NH) for library construction and sequencing. The Illumina DNA Prep Tagmentation Kit was used to prepare the DNA for sequencing on the Illumina NextSeq 2000 platform (2 × 150 bp reads). Sequencing produced 2,337,174 reads for the *E. hormaechei* UMB11011A strain and 4,200,970 for *P. rettgeri* UMB11011B. Raw reads were assembled using the Bacterial and Viral Bioinformatics Resource Center (BV-BRC) website v3.35.5 (10) with the “auto” parameter. BV-BRC first trimmed the raw reads using Trim Galore v0.6.5 (https://github.com/FelixKrueger/TrimGalore), assembled the reads using Unicycler v0.4.8 (11), and polished the assembly with Pilon v1.23 (12). Genome coverage, completeness, and contamination were computed by BV-BRC. Genome annotations were produced using the National Center for Biotechnology Information Prokaryotic Genome Annotation Pipeline v6.7 (13).

Table 1 lists the genome statistics for these two strains. These two genomes increase the number of sequenced strains for these two species, which are currently underrepresented in the LUEREC collection (PRJNA316969 and PRJNA970254) and the catalog of the urinary microbiota (14). Additional sequencing of urinary isolates for these two species is needed to gain insight into their genetic diversity and putative role in the urinary community.

**TABLE 1:** Genome assembly statistics

Strain	UMB11011A	UMB11011B
Species	*E. hormaechei*	*P. rettgeri*
SRA accession no.	SRR28710903	SRR28710899
Assembly accession no.	JBCGEE000000000	JBCGED000000000
No. of raw reads	2,337,174	4,200,970
Assembly length (bp)	4,864,048	4,591,325
G + C (%)	55.21	40.23
No. of contigs	47	36
Contigs N50 (bp)	320,976	263,088
Coverage (x)	59.54	134.89
Completeness (%)	96.5	100
Contamination (%)	0	0.7

## Data Availability

Table 1 lists the SRA and assembly accession numbers for the two strains.
